# Early detection of myocarditis caused by immune checkpoint inhibitor therapy with nivolumab and ipilimumab for advanced recurrent renal cell carcinoma

**DOI:** 10.1007/s00262-025-03945-0

**Published:** 2025-02-04

**Authors:** Maki Todo, Yodo Gatate, Shintaro Nakano, Go Kaneko, Masayuki Hagiwara, Takayuki Takahashi, Yuta Umezawa, Genji Ueda, Shiho Ishikawa, Yoshinori Makino, Masafumi Oyama, Suguru Shirotake

**Affiliations:** 1 Department of Pharmacy, Saitama Medical University Saitama International Medical Center, 1397-1, Yamane, Hidaka, Saitama, 350-1298 Japan; 2 Department of Cardiology, Saitama Medical University Saitama International Medical Center, Saitama, Japan; 3 Department of Uro-Oncology, Saitama Medical University Saitama International Medical Center, Saitama, Japan

**Keywords:** Nivolumab and ipilimumab, Renal cell carcinoma, Myocarditis, High-sensitivity Troponin, Onco-cardiology team

## Abstract

Combination therapy with nivolumab and ipilimumab for advanced renal cell carcinoma (RCC) may cause immune-related myocarditis; however, its incidence in this cancer type and regimen remains unknown. At our institution, we measure biomarkers, such as high-sensitivity Troponin (hsTn), and perform electrocardiograms (ECGs) and echocardiography before and every month after the initiation of this therapy, and the findings obtained and patients’ symptoms are continuously monitored by physicians and pharmacists. A retrospective survey was conducted on physiological and biochemical test findings and immune-related adverse events in patients with advanced RCC who received combination therapy with nivolumab and ipilimumab between October 1, 2018 and December 31, 2023. Patients suspected of having myocarditis consulted with cardiologists. Myocarditis due to this therapy was detected in 5 of the 86 patients (5.8%) assessed using the European Society of Cardiology 2022 guidelines. There were no fatal symptoms or death due to myocarditis. The median time to the onset of myocarditis was 25 days (21–86 days). The early detection of myocarditis caused by this therapy requires the monitoring of changes by periodically measuring hsTn and other cardiac markers and performing ECGs and echocardiography from the early stages of administration through to the end of treatment. In addition to checking symptoms, if these abnormalities are detected and myocarditis is suspected, prompt collaboration with cardiologists is recommended. Our management strategy of care by a onco-cardiology team may contribute to the early diagnosis and treatment of myocarditis.

**Supplementary Information:**

The online version contains supplementary material available at 10.1007/s00262-025-03945-0.

## Introduction

Combination therapy with nivolumab and ipilimumab, which are immune checkpoint inhibitors (ICIs), is the first-line treatment for intermediate- and poor-risk groups in the International Metastatic Renal Cell Carcinoma Database Consortium (IMDC) classification for advanced renal cell carcinoma (RCC) [1]. While the treatment efficacy of ICIs has been demonstrated, a wide range of immune-related adverse events (irAEs) have been reported, including hepatitis, colitis, myocarditis, skin disorders, fulminant type 1 diabetes, adrenal insufficiency, interstitial lung disease, and severe myasthenia gravis [2–6]. Among myocarditis cases, severe and fatal clinical courses have been reported [7, 8].

Survey of Bristol-Myers Squibb post-marketing safety databases reported that myocarditis was more frequent and severe in patients receiving ICI combination therapy than in those receiving ICI monotherapy (0.27 vs. 0.06%, *P* < 0.001, 5 fatal events vs. 1 fatal event) [7]. A retrospective study using a database subsequently reported an incidence of 0.41% for ICI monotherapy and 1.33% for ICI combination therapy [9]; therefore, ICI combination therapy is regarded as a risk factor for myocarditis. In addition, a multicenter retrospective registry study conducted overseas showed that the incidence of myocarditis including both ICI monotherapy and ICI combination therapy was 1.14% [10]. These studies [7–10] included multiple or other cancer types and did not specifically focus on the frequency of therapy with nivolumab and ipilimumab for advanced RCC.

ICI-myocarditis is the most fatal irAE with an estimated mortality rate of 25–60% [10–15]. In patients receiving combination therapy with nivolumab and ipilimumab, myocarditis was diagnosed a median of 17 days after the first treatment (range, 13–64 days) [7], and there was also a risk of its early onset.

Combination therapy with nivolumab and ipilimumab as the untreated first-line setting is currently used not only for RCC, but also for non-small cell lung cancer [16], malignant pleural mesothelioma [17], malignant melanoma [18], and esophageal cancer [19]. Dosage and administration schedules differ across cancer types, potentially affecting the incidence of myocarditis. Although there are case reports of myocarditis caused by ICI combination therapy for RCC [20, 21], there are currently no survey reports on the incidence of myocarditis other than case reports. Combination therapy with nivolumab and ipilimumab as the first-line treatment for advanced RCC is associated with a high risk of irAEs, particularly myocarditis; however, its incidence in this cancer type and regimen remains unknown.

Current domestic and international guidelines for ICIs highlight the importance of blood tests, particularly myocardial troponin, as a rapid, specific, and highly sensitive tool for diagnosing myocarditis. To diagnosis myocarditis, domestic and international guidelines recommend blood tests for biomarkers, such as cardiac troponin, electrocardiograms (ECGs), echocardiograms, and cardiovascular cardiac magnetic resonance (CMR) [2–6].

When this treatment began in 2018, specific details, such as the intervals and frequency of screening tests, had not been established. At our institution, we established a collaborative system between cardiologists and oncology pharmacists (onco-cardiology team) to develop screening test protocols and coordination, conducting regular screening tests from before to during treatment since the introduction of ICI therapy. The European Society of Cardiology (ESC) Guidelines were published in 2022, and the protocol used in our institution closely aligns with these guidelines, particularly in conducting regular and sensitive tests, such as troponin, a key myocardial biomarker.

The present study investigated the incidence of myocarditis in patients receiving combination therapy with nivolumab and ipilimumab as a first-line treatment for advanced RCC at our institution.

## Patients and methods

### Subjects and survey period

This study focused on patients diagnosed with advanced RCC who received combination therapy with nivolumab and ipilimumab as the standard treatment regimen. Therapy was continued until disease progression or the occurrence of intolerable irAEs. Subjects included patients whose treatment started at Saitama Medical University Saitama International Medical Center, Saitama, Japan between October 1, 2018 and December 31, 2023. The survey period was until June 30, 2024.

### Diagnostic criteria for myocarditis

The definition of myocarditis was based on the ESC Guidelines [5]. An excerpt from these guidelines is provided below.

The diagnosis of ICI myocarditis is as follows: Pathohistological diagnosis: Multifocal inflammatory cell infiltrates with overt cardiomyocyte loss by light microscopy, Clinical diagnosis: Troponin elevation (new or significant change from baseline) with 1 major criterion or 2 minor criteria, after exclusion of acute coronary syndromes and acute infectious myocarditis based on clinical suspicion. Major criterion: CMR diagnostic for acute myocarditis (modified Lake Louise criteria [22]). Minor criteria: Clinical syndrome (including any one of the following: fatigue, myalgias, chest pain, diplopia, ptosis, shortness of breath, orthopnea, lower-extremity edema, palpitations, light-headedness/dizziness, syncope, muscle weakness, cardiogenic shock), Ventricular arrhythmia (including cardiac arrest) and/or new conduction system disease, Decline in left ventricular (LV) systolic function, with or without regional wall motion abnormalities in a non-Takotsubo pattern, Other immune-related adverse events, particularly myositis, myopathy, myasthenia gravis, are suggestive of CMR.

In the present study, a decline in left ventricular (LV) systolic function was defined as “a decrease in LVEF > 10 percentage points, to a value < 53%” based on the American Society of Echocardiography and the European Association of Cardiovascular Imaging [23].

According to these guidelines, myocardial biopsy is performed on cases in which myocarditis is suspected at the discretion of cardiologists [5, 6]. The effectiveness of treatment for myocarditis is assessed by a cardiologist based on survival, biomarker testing, ECGs, echocardiograms, and CMR.

### Survey items

The following items from electronic medical records were retrospectively examined.

1. Patient characteristics: RCC IMDC classification, age, sex, estimated glomerular filtration rate (eGFR), presence or absence of nephrectomy, dialysis, medical histories or comorbidities (hypertension, dyslipidemia, obesity, current smoking, diabetes mellitus, and previous cerebrovascular/ heart disease)
2. Results of physiological and biochemical tests before and after administration: ECG abnormalities, left ventricular ejection fraction (LVEF) (%), positive for high-sensitivity Troponin (hsTn) (pg/mL), creatine kinase (CK) (U/L), and brain natriuretic peptide (BNP) (pg/mL). Positivity for hsTnI was defined as a value above the upper limit of normal (ULN) (26.2 pg/mL). hsTnI was measured by Abbott Architect (Abbott Diagnostics, Abbott Park, IL, USA). Positivity for hsTnT was defined as a value above the ULN (14.0 pg/mL). hsTnT was measured by Roche Diagnostics (Roche Diagnostics, Japan). The troponin result was considered to be positive if it exceeded the 99th percentile of the normal reference range. hsTn measured in this institution was changed from hsTnI to hsTnT in April 2022. At our facility, echocardiography was performed in accordance with the internal rule that the Teichholz method is used to assess cardiac function when it is normal, while Simpson’s method [24, 25] is reserved for cases where cardiac dysfunction is suspected. Therefore, LVEF is higher with the Teichholz method than with Simpson’s method, and LVEF is presumed to be above the normal range. In all patients with impaired LVEF, the urologic oncologist consulted with cardiologists, and treatment was initiated after discussions. ECG abnormalities were judged by cardiologists, and then divided into major abnormalities according to the guidelines of the Japan Society of Ningen-Dock, which are broadly used for screening settings in Japan [26, 27].
3. Number of patients with new abnormally elevated hsTn after administration and underlying reasons. A new abnormal elevation in hsTn was defined as a value above the ULN (hsTnI: 26.2 pg/mL, hsTnT: 14.0 pg/mL) and more than the baseline value of the patient.
4. In patients diagnosed with myocarditis, the time of onset, changes in ECG at onset, hsTn (pg/mL), LVEF (%), CK (U/L), BNP (pg/mL), whether myocardial biopsy was performed, symptoms at the onset of myocarditis, the total number of treatment courses with nivolumab and ipilimumab, whether steroid treatment was used, other cardiovascular therapeutics, whether ICIs were re-administered as a subsequent treatment for RCC, survival, and the next therapy were examined.
5. Number of patients with irAEs diagnosed as ≥ Grade 3, except for myocarditis. Regarding the evaluation method for AEs, except for myocarditis, the Common Terminology Criteria for Adverse Events ver. 5.0 (CTCAE ver. 5.0) [28] was used to assess the severity of AEs.

### Screening protocols and collaboration system

**Fig. 1:**
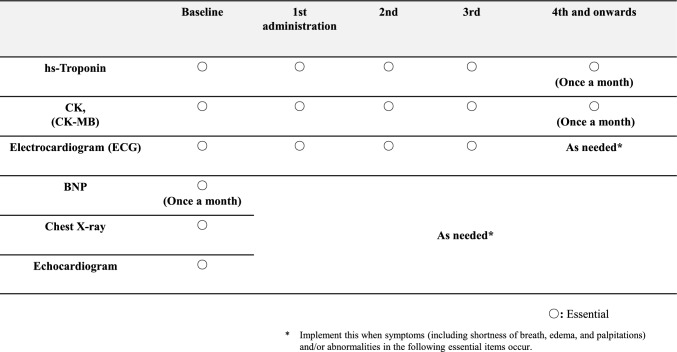
The routine screening test protocol

**Fig. 2:**
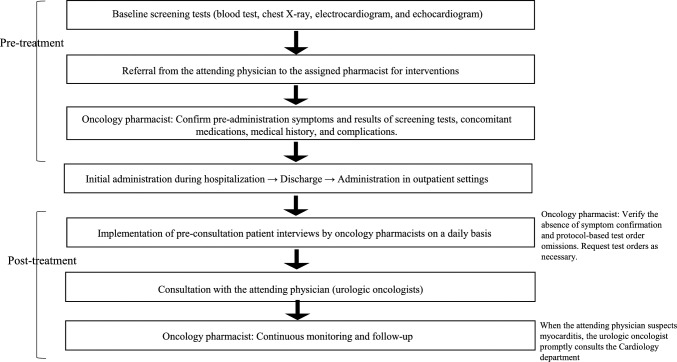
Collaborative care system involving urologic oncologists, oncology pharmacists, and cardiologists

The screening protocol was implemented for all cases and recommendations from specialized cardiologists. The administration interval was 21 days, with a 14 (or 28)-day interval for switching to nivolumab monotherapy. Screening tests for hsTn, CK, and BNP were performed before and approximately monthly after the initiation of administration. ECGs were conducted from baseline to the third administration. Additional tests were performed when abnormalities were detected during this period, as outlined in Fig. 1. The collaboration system between physicians (urologic oncologists) and oncology pharmacists is shown in Fig. 2. Oncology pharmacists (belong to the onco-cardiology team) worked in collaboration with physicians to monitor all cases throughout the treatment period, including confirming results of screening tests and symptoms before and after administration, confirming medical histories and comorbidities. Oncology pharmacists also educated patients on seeking medical attention when symptoms appeared. If myocarditis was suspected, the patient was promptly referred to cardiologists (onco-cardiology team).

### Statistical analysis

Comparisons of patients diagnosed with and without myocarditis were performed using the *t*-test for a parametric analysis and the Mann–Whitney U-test or Fisher’s exact probability test for a nonparametric data analysis. Statistical analysis were conducted using IBM SPSS Statistics for Windows, version 27.0 (IBM Inc., USA). A *p* value less than 0.05 was considered to be significant.

## Results

### Patient characteristics

**Fig. 3:**
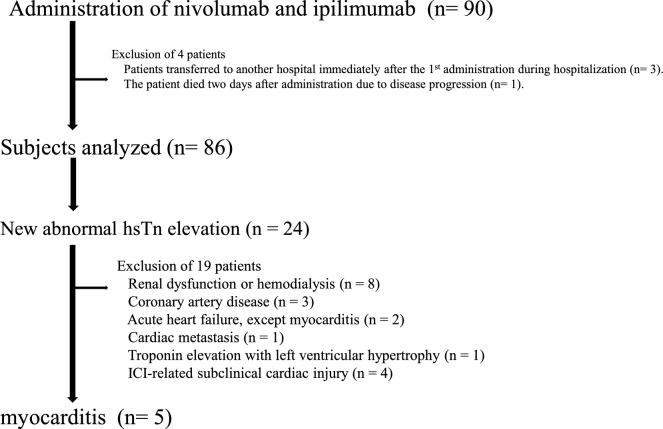
Consort diagram

Combination therapy with nivolumab and ipilimumab was administered to a cohort of 90 patients, with 86 being included in subsequent analyses (Fig. 3). In the IMDC classification, there were no cases in the Favorable-risk group, 47 (54.7%) in the Intermediate-risk group, and 39 (45.3%) in the Poor-risk group. Patient backgrounds, medical histories, complications, physiological tests before administration, and biochemical test results based on the presence or absence of myocarditis are shown in Table 1. No factors related to the occurrence of myocarditis were identified.

**Table 1:** Patient backgrounds and physiological and biochemical test results before administration

Before administration	Myocarditis (+) (*n* = 5)	Myocarditis (−) (*n* = 81)	*P* value
Age (median) (range)	68.0 (57–74)	67.0 (32–87)	0.698^a^
Sex (male/female, *n*) (%)	(5/0) (100/0)	(56/25) (69.1/30.9)	0.315^b^
Implementation of nephrectomy (yes/no, *n*) (%)	(3/2) (60/40)	(25/56) (30.9/69.1)	0.324^b^
Hemodialysis (yes/no, *n*) (%)	(0/5) (0/100)	(5/76) (6.2/93.8)	1.000^b^
eGFR (median) (range)	62.2 (45.0–115.2)	57.3 (4.1–145.5)	0.347^a^
Comorbidities (*n*, (%))			
Hypertension	5 (100)	53 (65.4)	0.168^b^
Dyslipidemia	3 (60)	34 (42.0)	0.648^b^
Obesity	2 (40)	24 (29.6)	0.636^b^
Current smoking	2 (40)	24 (29.6)	0.636^b^
Diabetes mellitus	0 (0)	22 (27.2)	0.322^b^
Previous cerebrovascular/heart disease	0 (0)	14 (17.3)	0.586^b^
Baseline physiological and biochemical test results			
*Abnormal ECG present (yes/no, *n*) (%)	(1/4) (20/80)	(14/67) (17.3/82.7)	1.00^b^
Atrial fibrillation	0	3	
Sinus tachycardia (≥ 101 bpm)	0	4	
Abnormal Q wave	0	5	
High left ventricular voltage (with a ST-T change)	0	3	
Poor R progression	1	2	
LVEF (median (%)) (range)	73.0 (47–76)	68.0 (32–89)	0.536^a^
Troponin-positive (yes/no, *n*) (%)	(1/4) (20/80)	(6/75) (7.4/92.6)	0.353^b^
CK (median, (U/L)) (range)	38.0 (7.0–97.0)	49.0 (5.0–228.0)	0.651^a^
BNP (median, (pg/mL)) (range)	16.1 (7.3–69.7)	32.6 (5.8–652.7)	0.184^a^

^a^The Mann–Whitney *U*-test, ^b^ Fisher’s exact probability test. * ECG abnormalities were divided into major abnormalities according to the guidelines from the Japan Society of Ningen-Dock, which are broadly used for screening settings in Japan

### Number of patients with new abnormal elevations in hsTn after administration and underlying reasons

Patients with elevated hsTn levels at baseline and above the reference value were extracted. These patients were closely monitored during treatment. Twenty-four patients (27.9%) had elevated hsTn levels. The reasons for elevations are shown in Fig. 3. Five patients were diagnosed with myocarditis.

### Onset of myocarditis

Myocarditis was diagnosed by cardiologists in 5 of the 86 patients (5.8%). Details on the post-administration outcomes of the 5 patients diagnosed with myocarditis are shown in Table 2. No serious or fatal cases of myocarditis were observed. There were 5 males. The median age of patients (range) was 68 years (57–74 years). The median onset was 25 days (21–86). Three of the 5 patients (patient Nos. 1, 2, and 3) had multiple irAEs.

**Table 2:** List of 5 patients diagnosed with myocarditis

Patient No Sex Age	another irAEs	Number of days from the initial administration to the onset	Baseline ECG findings	Newly emerged ECG abnormalities	baseline hsTn (pg/mL)	hsTn-diagnosed myocarditis (pg/mL)	CK baseline/peak (U/L)	LVEF baseline/diagnosed myocarditis (%)	BNP Baseline/diagnosed myocarditis (pg/mL)	Cardiac biopsy/pathology results	Symptoms	Total courses of treatment with nivolumab and ipilimumab	Treatment outcomes	Corticosteroid therapy	Other cardiovascular therapeutics	Survival	Next therapy
No. 1 Male 61	Skin disorder (TEN) (Grade 4)	26	Left QRS axis deviation	Sinus tachycardia (102 bpm)	< 5.0 (hsTnI)	88.8 (hsTnI)	38/92	73/51	6/131.5	–	Fever (37.8°C) rash (Grade 4) tachycardia (Grade 1) fatigue (Grade 1)	1	Discontinuation	Methylprednisolone (1000 mg div)	–	Cancer death	Cabozantinib, best supportive care
No. 2 Male 68	Sialadenitis (Grade 3) Pancreatitis (Grade 3)	21	Normal	None	< 5.0 (hsTnI)	231.1 (hsTnI)	24/11	76/67	35.1/60.6	–	Anorexia (Grade 2) taste disorder (Grade 2) fatigue (Grade 1)	4	Temporary suspension, Rechallenge (nivolumab only)	Prednisolone (10 mg oral)	–	Cancer death	Cabozantinib, best supportive care
No. 3 Male 68	Pituitary adrenal insufficiency (Grade 3) Destructive thyroiditis (Grade 1)	86	Normal	QT prolongation	23.4 (hsTnI)	159.2 (hsTnI)	97/387	70/69	7.3/5.8	–	Anorexia (Grade 2) fatigue (Grade 2) cardiac syncope (Grade 3)	4	Discontinuation	Hydrocortisone (100 mg div)	–	Survival	No treatment, observation
No. 4 Male 74	–	25	Normal	Complete right bundle branch block	11.2 (hsTnT)	192.0 (hsTnT)	74/950	75/62	16.1/9.6	Performed/Myocarditis	Tachycardia (Grade 1)	1	Discontinuation	Methylprednisolone (1000 mg div) co-administration of immunoglobulins	–	Survival	Axitinib
No. 5 Male 57	–	21	Poor R progression	Ventricular conduction disorder	17.6 (hsTnT)	44.5 (hsTnT)	7/14	47/31	69.7/456.5	Performed/Myocarditis	shortness of breath (Grade 1)	2	Discontinuation	–	Oral therapy Enalapril Carvedilol Furosemide Spironolactone	Survival	No treatment, observation

The grades of adverse events, except for myocarditis, were selected according to Common Terminology Criteria for Adverse Events (CTCAE) version 5.0.‘–’ indicates that it has not been implemented

No. 2 patient: LVEF (%) that diagnosed myocarditis was 67 with wall motion abnormality

TEN: Toxic epidermal necrolysis

Only one case (patient No. 5) had abnormal ECG (Poor R progression), LVEF < 50%, and positive hsTn (< 20 pg/mL) prior to administration. In one case (patient No. 3) diagnosed with myocarditis, pituitary-adrenal insufficiency was detected and, thus, hydrocortisone pulse therapy was promptly administered, which prevented the need for myocardial biopsy. Myocarditis improved with hydrocortisone treatment. One case (patient No. 4) had a complete right bundle branch block and prednisolone pulse treatment was started early. However, hsTn decreased in the remaining patient (patient No. 5), and symptoms improved after the suspension of treatment; therefore, steroid treatment was not started. Myocarditis improved in all patients who received corticosteroid treatment. ICIs were not re-administered to 4 of the 5 patients with myocarditis. One case (Patient No. 2) was re-administered nivolumab only with careful ongoing cardiac monitoring after corticosteroid treatment. Two of the 5 patients had CK levels above the reference value: Patient No. 3 and Patient No. 4, who newly developed a right bundle branch block, and high-dose steroids were started for these patients.

**Fig. 4:**
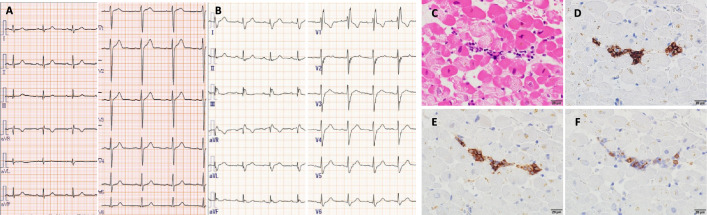
ECGs and pathological findings of myocarditis. A 74-year-old male (No. 4) with RCC treated with nivolumab and ipilimumab. **A** Before the administration of nivolumab and ipilimumab, findings were mostly within normal ranges. **B** After the administration of nivolumab and ipilimumab, with a newly emerged right bundle branch block. **C** At myocardial biopsy, cardiac tissue stained with hematoxylin and eosin showed myocyte degeneration accompanied by a mononuclear cell infiltrate. The marked infiltration of T cells was also noted, as shown by immunostaining for CD3 (**D**). This infiltrate included approximately equal percentages of CD4 (**E**) and CD8 (**F**) T cells. Scale bar: 20 μm. CD: cluster of differentiation

Of the 5 patients diagnosed with myocarditis, 3 survived and 2 died, but not due to myocarditis. Two of the 3 surviving patients (patient Nos. 3 and 5) are undergoing follow-up observations without treatment because RCC has not progressed. Treatment for the other patient (patient No. 4) was changed to the oral multikinase inhibitor axitinib due to myocarditis. Two patients who died (patient Nos. 1 and 2) were receiving best supportive care due to disease progression after treatment with cabozantinib. The ECGs and pathological findings of a 74-year-old male patient (No. 4) are shown in Fig. 4.

### Number of patients with irAEs ≥ Grade 3, except for myocarditis

Table 3 shows a list of patients diagnosed with Grade 3 or higher irAEs caused by this treatment. All patients were administered steroid treatment and improved, and no deaths due to irAEs were reported.

**Table 3:** List of immune-related adverse events (irAEs) diagnosed as ≥ Grade 3, except for myocarditis

Immune-related adverse events (irAEs)	*n* (%)
Pituitary adrenal insufficiency	19 (22.1)
Skin disorder	9 (10.5)
Hepatitis	7 (8.1)
Pancreatitis	5 (5.8)
Colitis	5 (5.8)
Joint pains	3 (3.5)
Sialadenitis	3 (3.5)
Type 1 diabetes	2 (2.3)
Nephritis	2 (2.3)
Uveitis, Harada’s syndrome	2 (2.3)
Fever	1 (1.2)
Gastritis	1 (1.2)
Pemphigus vulgaris	1 (1.2)
Scleroderma	1 (1.2)
Pneumonia	1 (1.2)

AEs were graded according to the National Cancer Institute Common Terminology Criteria for Adverse Events (CTCAE) ver.5.0

## Discussion

This study provides insights into the incidence of myocarditis based on the regular screening of patients treated with nivolumab and ipilimumab for advanced RCC in clinical settings. To the best of our knowledge, few studies reported that myocarditis was caused by combination therapy with nivolumab and ipilimumab for advanced RCC in 5 out of 86 patients assessed using the ESC 2022 guidelines [5], with an incidence of 5.8%. None of these cases progressed to fatal symptoms or death. We implemented an institutional regular screening test protocol in all patients, and also ensured that the protocol was administered with the intervention of oncology pharmacists from the onco-cardiology team, who continued to closely monitor patients’ symptoms and test findings. This resulted in a mortality rate of 0% due to the suspension of treatment with ICIs at the appropriate point in time and prompt consultation with cardiologists, as well as the early diagnosis and treatment of myocarditis before it became severe. Our management strategy of care by an onco-cardiology team may contribute to the early diagnosis and treatment of myocarditis. Cabozantinib [29, 30], an oral multikinase inhibitor, is recommended as a second-line treatment for RCC when ICIs cannot be used due to irAEs after nivolumab and ipilimumab. Therefore, if the therapeutic benefit of oncologic therapy for RCC is required, as in the present cases, switching to these agents needs to be considered with continued cardiac monitoring after nivolumab and ipilimumab.

Screening for myocarditis was performed using blood biomarkers, symptoms, ECGs, and echocardiography in the present study. Power et al. [31] showed that the severity of myocarditis was associated with the magnitude of change in troponin (> 20- to 2000-fold higher than the ULN), thymoma, low-QRS voltage (EKG: Sokolow-Lyon voltage) ≤ 0.5 mV, depressed LVEF (< 50%), and cardio-muscular symptoms in patients with multiple cancer types using data collected retrospectively from a multicenter registry data from 17 countries including Japan, between 2014 and 2023 (*n* = 748). A previous study reported that troponin was elevated in more than 90% of patients with myocarditis [15], and the severity of myocarditis was associated with troponin levels [13, 31]. In all 5 patients in this study, hsTn was within 15-fold of the ULN. Furthermore, one patient with LVEF < 50% (patient no. 5) and another patient with syncope (patient no. 3) met these criteria for severity. ECGs showed “new conduction system disease” as described in the ESC 2022 guidelines [5] in two patients: one patient (patient no. 4) with a complete right bundle branch block and one patient (patient no. 5) with a ventricular conduction disorder, but no ventricular arrhythmia. A quantitative analysis of ECGs in patients diagnosed with myocarditis is shown in Table 1 in Supplement 1. ICI-associated myocarditis in most cases is accompanied by ECG changes [15]. Power et al. [32] showed that ECGs in ICI-myocarditis with ventricular tachycardias, heart block, and low-voltage and pathological Q waves were associated with myocarditis-related mortality and life-threating arrhythmia. The presence of a high frequency of serious conduction disturbances may be associated with T cell-mediated cytotoxicity, which affects the cardiac conduction system. Concomitant myositis or myasthenia gravis was not observed in the present study. BNP was elevated in 3 of the 5 patients examined herein. N-terminal pro-brain natriuretic peptide levels were increased in 66 and 77.5% of myocarditis cases in two previous studies [10, 15]; however, they may also be elevated in many cancer patients due to inflammation associated with cancer. Therefore, this result was not specific to ICI-myocarditis. As for LVEF, Zhang et al. [33] reported that left ventricular function was preserved in more than half (61%) of patients pathologically diagnosed with ICI-myocarditis. Although one case (patient No. 4) in the present study belonged to this category, the diagnosis requires a comprehensive judgment. In addition, previous studies reported elevated levels of troponin and CK in patients with myocarditis [34], and Furukawa et al. [35] showed that elevated CK preceded elevated Troponin-I in patients with moderate to severe symptoms of myocarditis, while elevated CK was not observed in asymptomatic or mildly ill patients, suggesting that elevated CK is an early biomarker of severe myocarditis. In the present study, although the time lag between hsTn and CK was not confirmed, the results obtained are considered to be similar. In addition, since myocarditis may occur concomitantly with myositis and myasthenia gravis [2–6], it is considered important to also monitor CK levels. The results shown in Table 2 suggest that cases of myocarditis in the present study represent a less severe population than in previous reports [13,31] of deaths, which indicate that myocarditis was treated before it became more severe.

A point of interest is the difference in the incidence of myocarditis between the present study and a previous retrospective analysis. There have been no similar reports of RCC using the same diagnostic criteria [5] as in this study, so incidence rates cannot be compared. Although the incidence of myocarditis was 0.27% to 1.33% with combination therapy with nivolumab and ipilimumab in the reports [7, 9, 10], it was high in the present study; however, there were no fatal cases. In addition, the diagnostic criteria is not the same, and Johnson et al. [7] had shown that in clinical trials, there was no routine testing for myocarditis by means of either biochemical analysis or cardiac imaging. Furthermore, a meta-analysis of randomized clinical trials confirmed that biomarkers, such as hsTn and ECGs, for cardiotoxicity were not routinely monitored during treatment, making it impossible to detect cardiotoxicity [36]. This may be because only severe and fatal cases were identified at that time, and asymptomatic or mild cases were ignored. One of the reasons for the increase in the incidence may be the implementation of regular screening.

In this study, steroid treatment was administered early along with a prompt diagnosis in accordance with the guidelines [2–6], which prevented the condition from worsening.

In the 5 cases of myocarditis in the present study, some developed without any subjective symptoms specific to myocarditis alone, and asymptomatic myocarditis has also been reported [35, 37, 38]. As shown in Tables 2 and 3, a wide variety of irAEs occur with nivolumab and ipilimumab, including multiple irAEs. Although all patients were monitored during treatment, it was difficult to extract symptoms specific to myocarditis alone. Patients with advanced or recurrent cancer develop a number of symptoms due to metastasis and disease progression. Furthermore, the onset of myocarditis occurred on the 21st day in 2 cases and on the 17th day in another case [7], which is an early stage; therefore, it is possible that testing after symptoms appear may delay detection. Therefore, in ICI combination therapy, which increases the frequency and severity of myocarditis, it is necessary to regularly test hsTn and other biomarkers and perform ECG and echocardiography before and during treatment prior to the appearance of symptoms, rather than testing after symptoms are confirmed, in order to reduce its severity and mortality rate.

The pharmacists requested orders from the physician for biomarker tests and ECGs when they were deemed necessary or when there were indications of omission. They also identified patients suspected of having myocarditis due to abnormal test results and facilitated referrals to cardiology specialists. This may have contributed to the early diagnosis, treatments, increasing diagnostic rates of myocarditis and, as a result, its progression and death were avoided. In addition, the incidence of irAEs is high with ICI combination therapy, and multiple irAEs may appear. Therefore, the regular symptom monitoring of patients by a team including oncology pharmacists and other medical staff, as described herein, is important for the early detection and treatment of irAEs, including myocarditis.

Elevated hsTn levels were observed in 24 out of 86 cases (27.9%). As shown in Fig. 3, 8 of the 24 cases were attributed to decreased renal function or dehydration. hsTn levels are considered to increase in patients with chronic renal failure, those receiving dialysis, and dehydration associated with renal as well as myocardial dysfunction, which decreases the clearance of hsTn [39, 40, 41]. The present study targeted patients with RCC, including patients with one kidney who had undergone nephrectomy, patients with renal dysfunction, patients receiving dialysis, and elderly patients. It is also necessary to understand the significance of elevated hsTn levels and to make appropriate differential diagnoses.

The present results showed that 5 patients with myocarditis had medical histories or complications that were risk factors for cardiovascular diseases, such as hypertension and dyslipidemia; however, no clear contributing factors were found. Risk factors for RCC and cardiovascular diseases are the same, such as smoking and obesity [42]. Since factors related to the development of ICI-associated myocarditis in patients with RCC have not been reported, further studies are warranted.

In addition, foreign guidelines [5, 6] state that a diagnosis by CMR is required as a diagnostic criterion for ICI myocarditis; however, CMR was not performed on any cases at our institution.

This study was limited in that it was a single-center study that only examined a small number of patients. In our institutional practice, Simpson’s method to measure LVEF is only used for patients with abnormal left-ventricular motion observed in a visual estimation, which may lead to an inaccurate assessment of patients without obvious dysfunction. Furthermore, CMR and myocardial biopsy are not performed on all patients, potentially affecting the incidence of myocarditis. In Japan, routine CMR testing for all patients is challenging, and myocardial biopsy is also difficult due to the prioritization of cancer patients’ conditions. The first improvement is the unification of Simpson’s method for the LVEF evaluation, while the second improvement is considering CMR or myocardial biopsy based on the patient’s condition. Therefore, the results obtained herein need to be confirmed in future studies on larger prospective cohorts in consideration of these points.

## Conclusion

This study provides insights into the incidence of myocarditis based on the regular screening of patients treated with nivolumab and ipilimumab for advanced RCC in clinical settings. None of the cases progressed to fatal symptoms or death. Based on our results, we propose that cardiovascular screening should be performed after ICIs administration, and recommended the management by an onco-cardiology team. Further studies are needed for patient risk stratification and to validate this method of monitoring patients.

## Supplementary Information

Below is the link to the electronic supplementary material.

Supplementary file 1 (DOCX 16 KB)

## Data Availability

No datasets were generated or analyzed during the current study.

## Abbreviations

BNP: Brain natriuretic peptide
CD: Cluster of differentiation
CK: Creatine kinase
CMR: Cardiac magnetic resonance
CTCAE: Common Terminology Criteria for Adverse Events
ECGs: Electrocardiograms
eGFR: Estimated glomerular filtration rate
ESC: European Society of Cardiology
hsTn: High-sensitivity Troponin
hsTnI: High-sensitivity Troponin-I
hsTnT: High-sensitivity Troponin-T
ICIs: Immune checkpoint inhibitors
IMDC: International Metastatic Renal Cell Carcinoma Database Consortium
irAEs: Immune-related adverse events
LV: Left ventricular
LVEF: Left ventricular ejection fraction
RCC: Renal cell carcinoma
ULN: Upper limit of normal
TEN: Toxic epidermal necrolysis
