# Supplementary material for: Early detection of myocarditis caused by immune checkpoint inhibitor therapy with nivolumab and ipilimumab for advanced recurrent renal cell carcinoma
Source: Cancer Immunol Immunother. 2025 Feb 4;74(3):97. doi: 10.1007/s00262-025-03945-0 (PMC11794898; doi:10.1007/s00262-025-03945-0)
Supplement: Supplementary file 1 — (DOCX 16 KB) [file 262_2025_3945_MOESM1_ESM.docx]

| **Table 1 in Supplement 1 Quantitative analysis of ECGs before and after administration (n = 5)** | | | |
| --- | --- | --- | --- |
|  | Before administration | After administration | *P value* |
|  | mean (SD) | mean (SD) |  |
| Heart rate (beats/min) | 79.8 (6.4) | 85.60 (9.81) | 0.289 ^a^ |
| RR interval (ms) | 754.6 (59.9) | 708.8 (73.3) | 0.311 ^a^ |
| PR interval (ms) | 170.0 (27.1) | 178.8 (31.8) | 0.113 ^a^ |
| QRS time (ms) | 107.2 (6.9) | 119.8 (18.0) | 0.186 ^a^ |
| QT (ms) | 374.0 (11.0) | 379.8 (50.9) | 0.787 ^a^ |
| QRS axis (º) | 21.80 (37.0) | 36.60 (49.2) | 0.244 ^a^ |
| SV1 (mV) | 0.94 (0.40) | 0.70 (0.33) | 0.281 ^a^ |
| RV5 (mV) | 1.60 (0.70) | 1.61 (1.23) | 0.974 ^a^ |
| SV1+RV5 (mV) | 2.53 (0.45) | 2.30 (1.26) | 0.655 ^a^ |
| QTcB (ms) | 431.2 (17.4) | 451.3 (50.4) | 0.269 ^a^ |
| QTcF (ms) | 411.1 (13.1) | 426.0 (49.9) | 0.429 ^a^ |
|  | n (%) | n (%) | *P value* |
| Rhythm = sinus rhythm | 5 (100.0) | 5 (100.0) | 1.000 ^b^ |
| RR, PR, and QRS intervals were measured in lead II to the nearest 2 ms from the average. Heart rate was calculated using the 1/RR interval. The QT interval was measured in lead II or V5 (whichever provided the best delineation of the T wave). The highest value was quoted as the measured QT interval (QTm). Using the preceding RR interval, the corrected QT interval (QTc) was obtained by Bazett’s formula (QTcB = QTm/√RR) and Fridericia’s formula (QTcF = QTm/√RR^3^). R- and S-wave amplitudes were measured to the nearest µV as the mean of the highest amplitudes of QRS complexes; Sokolow-Lyon voltage criteria were used to define LVH (S wave in V1 + R wave in V5 or V6, whichever was greater).  QTcB: corrected QT interval obtained by Bazett’s formula; (QTcB = QTm/√RR)  QTcF: corrected QT interval obtained by Fridericia’s formula; (QTcF = QTm/√RR^3^)  SD: standard deviation.  ^a^ paired *t*-test, ^b^ Fisher’s exact probability test.  ms: millisecond | | | |
